# Operationalizing and validating disciplinary literacy in secondary education

**DOI:** 10.1007/s11145-018-9839-4

**Published:** 2018-03-21

**Authors:** Hiller A. Spires, Shea N. Kerkhoff, Abbey C. K. Graham, Isaac Thompson, John K. Lee

**Affiliations:** 1 Department of Teacher Education and Learning Sciences, North Carolina State University, 1890 Main Campus Dr., Raleigh, NC 27606 USA; 2 Department of Educator Preparation, Innovation and Research, University of Missouri-St. Louis, 1 University Blvd, St. Louis, MO 63121 USA

**Keywords:** Disciplinary literacy, Secondary education, Literacy in science, Literacy in mathematics, Literacy in history

## Abstract

The goal of this study was to define the construct and establish the validity of disciplinary literacy, which has recently gained attention from the implementation of the Common Core State Standards (National Governors Association Center for Best Practices & Council of Chief State School Officers in Common Core State Standards for English language arts & literacy in history/social studies, science, and technical subjects [PDF]. Authors, Washington, DC, 2010). After defining disciplinary literacy in the four core disciplines of English language arts, science, history and social studies, and mathematics, scales were developed and administered to a snowball sample of professionals nationwide, with 857 respondents. The data showed evidence of disciplinary literacy as a multidimensional construct with three related factors: source literacy, analytic literacy, and expressive literacy. Based on EFA and CFA results, we can conclude that there are at least three types of literacy in operation among the four core disciplines. The three factors of literacy varied significantly by the four core disciplines of English/language arts (ELA), science, history and social studies, and mathematics, supporting the notion that each discipline uses literacy uniquely. This is the first study of its kind to attempt to define, quantify, and validate the construct of disciplinary literacy.

## Introduction

Increasingly, educators have concerns with adolescent under-performance in literacy (Lee & Spratley, 2010). The Rand Reading Study Group (2002) found that reading comprehension is not improving among youth even though text complexity and technicality is on the rise. Likewise, the results for eighth and twelfth graders on the 2015 National Assessment of Educational Progress (NAEP) demonstrate troubling trends. Thirty-four percent of eighth graders scored at or above proficiency, and 24% performed below the basic level in reading. Only 4% of eighth grade students and 5% of twelfth grade students performed at the advanced level of proficiency (i.e., able to make connections across texts, evaluate and justify evidence, etc.). These low scores reflect a consistent trend in the last few decades and, in part, are impetus for the state and national standards’ shifts in English language arts (ELA), which focus on more rigorous investigations of complex text within the disciplines (e.g., National Governors Association Center for Best Practices [NGA Center] & Council of Chief State School Officers [CCSSO], 2010).

These concerns about reading achievement have ushered in new conversations about how to promote literacy within the disciplines (Draper & Siebert, 2010; Moje, 2002; Shanahan & Shanahan, 2008). Disciplinary literacy has been defined as “the use of reading, reasoning, investigating, speaking, and writing required to learn and form complex content knowledge appropriate to a particular discipline” (McConachie & Petrosky, 2010, p. 6). Shanahan and Shanahan (2012) suggested that disciplinary literacy is different from more traditional content literacy in that content area literacy focuses on strategies that struggling or novice readers can utilize when reading, while “disciplinary literacy emphasizes the unique tools that the experts in a discipline use to participate in the work of that discipline” (p. 2). Although the distinction between content literacy and disciplinary literacy is gaining ground within the field of literacy scholarship, the two terms are still sometimes used interchangeably (Shanahan & Shanahan, 2008).

Literacy demands increase as students progress through school. Some scholars say students are expected to evolve from decoding and fluency in early elementary grades to discipline-specific vocabulary and comprehension of informational texts in intermediate grades to disciplinary reading in secondary school and beyond. Therefore, a lack of intermediate reading proficiency in informationally-dense texts becomes a stumbling block to learning new content in all academic areas (Lee & Spratley, 2010; Shanahan & Shanahan, 2008; Snow & Moje, 2010; Spires, Kerkhoff, & Graham, 2016). In middle school, for example, students are developing disciplinary literacy, which should eventually include skills such as sourcing, close reading, and critical response to text (Shanahan, Shanahan, & Misischia, 2011; Graham, Kerkhoff, & Spires, 2017).

In order to increase reading achievement, the Carnegie Corporation as well as state and national standards have advocated that reading performance be supported across disciplines (e.g., ELA, science, history and social studies, and mathematics) rather than solely in the language arts classroom (Lee & Spratley, 2010; NGA Center & CCSSO, 2010). Current policy (i.e., state and national standards) is influencing disciplinary literacy practices in the classroom and instructional materials are being published on the topic. To date, qualitative research has described disciplinary literacy at the expert level. Research, however, has fallen short of establishing a measurable construct to operationalize the concept of disciplinary literacy in the secondary school context. In order to advance the research on disciplinary literacy, we need to validate the construct on the target population, which in relation to state and national standards, is secondary teachers. Validity is conditional. In fact, “a measure that is valid for one population may not be valid for another population (Vogt, King, & King, 2004, p. 240). Without an empirically valid construct, the field may be misapplying a construct from the expert context to the secondary context. It is important that the literacy field establish disciplinary literacy as an empirically valid construct generalizable to the secondary education context in order for research-based instructional practices to follow.

## Theorizing and conceptualizing disciplinary literacies

In order to understand disciplinary literacy both theoretically and conceptually, we chose to review the literature broadly and then narrow our focus on the four disciplinary domains that inform our research design. First, we explored discourse theory as an underlying tenet. Second, we explored disciplinary literacy as it is currently conceptualized within the field of secondary education (grades 6–12), with a focus on apprenticeship and problematizing the linear view of disciplinary literacy. Finally, we examined ELA, science, history and social studies, and mathematics and synthesized extant research that supports different literacies for each of these subject areas. This literature review served as the theoretical foundation on which to functionally examine how participants operationalize domain-specific literacy practices.

### Disciplinary literacy grounded in discourse theory

Middle and secondary literacy has been theorized from literacy perspectives rather than disciplinary perspectives. Moje (2008) advocated that using the tenets of the disciplines as a grounding tool has the potential to improve literacy practices. Similarly, we hold that the heart of disciplinary literacy is that each discipline has its own discourse. Discourse theory has been applied broadly across a range of fields including literacy, linguistics, and philosophy (Yang & Sun, 2010). We relied on Gee’s (1996) idea of literacy as “Discourse/s,” which depicts language as a means of identifying oneself as a member of a meaningful group or community. Gee (2011) synthesized ideas from across fields to create his term “discourse,” to signify everyday language in use, which is cultivated by cultural and societal contexts. This definition of literacy makes a distinction between lowercase *d* and capital *D* in order to demonstrate how people use language differently across contexts.

Snow and Uccelli (2009) synthesized the literature based on a variety of linguistic features pertinent to discourse in academic contexts. For example, within an academic context the language user is more likely to take an authoritative interpersonal stance rather than a situationally-driven stance. In terms of information load, the academic discourse user is prone to conciseness and density (Schleppegrell, 2001), rather than redundancy and sparsity. Lexical choice is another key feature of academic discourse, with an emphasis on high lexical diversity (Chafe & Danielewicz, 1987), formal expressions, and abstract or technical concepts.

Integral to disciplinary literacy and academic success is mastery of academic language; therefore, a discipline has a “need for precision through its texts or its language” (Johnson, Watson, Delahunty, McSwiggen, & Smith, 2011, p. 104). Each discipline has a unique language, and according to Johnson et al. (2011) each language is typically comprised of specialized vocabulary, sentence structure, and symbol systems. Thus, these different grammars or patterns of language include “differences not only in the nature of the technical vocabulary, but also in points of view, attribution of causation and agency, passive and active voice, and other linguistic differences that undergird the nature and purpose of the disciplines” (Shanahan & Shanahan, 2012, p. 10). Academic literacies require students to not only navigate abstract and technical words and concepts, but to do so across multiple contexts throughout their day (Bailey, 2007; Lea & Street, 2006). Academic language “stands in contrast to the everyday informal speech that students use outside the classroom environment” (Bailey, 2007, p. 12). These differences necessitate that students are aware of how these characteristics play out in the various disciplines and give students power and access to language by demystifying the way language is used in the disciplines. In this sense, academic discourse is contrasted with a deficit paradigm since students are empowered through learning the different uses of language in the appropriate contexts (Lea & Street, 2007).

### Current conceptions of disciplinary literacy

The literacy education field frames disciplinary literacy as a highly complex lens from which to view literacy practices. Some have called for moving the focus of secondary literacy to the disciplines (Moje, 2008) and discipline-specific literacy practices (Zygouris-Coe, 2012), while others call for maintaining a focus on general content-area strategies (Faggella-Luby, Graner, Deschler, & Drew, 2012; Heller, 2010). Meanwhile, others have called for a balanced approach that considers both content area and disciplinary literacy approaches (Brozo, Moorman, Meyer, & Stewart, 2013).

To date, disciplinary literacy has been theorized at the K-12 level (e.g., Ehren, Murza, & Malani, 2012; Fang & Coatoam, 2013; McConachie & Petrosky, 2010; Zygouris-Coe, 2012) more than it has been researched. Although philosophical and speculative discussions of this topic abound, empirical research has been slower to emerge at the K-12 level. The limited research on disciplinary literacy has taken various forms, including qualitative studies (e.g., Shanahan & Shanahan, 2008; Park, 2013; Pytash, 2012; Rainey & Moje, 2012), quantitative studies (e.g., Reisman, 2012), and mixed methods investigations (e.g., Achugar & Carpenter, 2012). Based on this literature, we conceptualized disciplinary literacy instruction as apprenticeship in the discipline through novice-expert relationships, where students development disciplinary literacy skills to knowledge construction in a discipline. We then discuss the problems with a strictly linear view of disciplinary literacy.

#### Apprenticeship

From an apprenticeship perspective, learning is situated and knowledge is constructed *in* one’s participation in a community of practice (Lave & Wenger, 1991). Participation in specific communities requires members to understand the various repertoires, routines, tools, vocabularies, and ideas that are used and uniquely situated in different communities. Above all, apprenticeship in the literacy of a discipline enables students to access knowledge in the different disciplines (McConachie & Petrosky, 2010; Moje, 2007). McConachie and Petrosky (2010) describe this model as “learning on the diagonal,” in which students actively and simultaneously display growth in their disciplinary habits of thinking and their content area knowledge. In the reading apprenticeship framework, Schoenbach, Greenleaf, and Murphy (2012) conceive of literacy through four overlapping dimensions: cognitive, social, personal, and knowledge building. Through growth in each of these dimensions, students develop new disciplinary identities of themselves as learners. Rather than reading *like* a historian or writing *like* a scientist, students take on the identity of a historian and of a scientist.

These disciplinary identities “are enactments of self that reflect the habits of mind, practices, and discourses—of the ways of knowing, doing and thinking and acting—associated with work in the disciplines” (Moje, 2011, p. 8). By and large, building disciplinary knowledge and identity is an intertwined and interactive process during which learning occurs from constructing knowledge and navigating different contexts (Moje, 2011). The purpose of this process is not necessarily for students to become disciplinary experts, but to expose them to the methods employed within the disciplines (Moje, 2015; Rainey & Moje, 2012). Disciplinary insiders not only participate in practices similar to other members of that community, but also can identify with other members and the knowledge that is associated with the discipline. Thus, a disciplinary insider must be able to engage in the cognitive, social, and semiotic processes specific to the discipline (Fang & Coatoam, 2013). Yet in order to apprentice learners toward mastery and participation as disciplinary insiders, there must be explicit instruction so that students can develop skills of the discipline over time.

#### Problematizing the linear view

The linear view asserts that reading skills develop hierarchically and requires students to progress developmentally from learning to read (i.e., basic literacy) to reading to learn (i.e., intermediate literacy; Chall, 1996; Shanahan & Shanahan, 2008). By the time students reach secondary school, language in the content areas becomes more technical and abstract. Students are expected to be able to read, write, think, and speak in the different disciplines, which requires them to understand the nuances that exist in the different subject areas and have strong foundational language skills (Ehren et al., 2012) as well as procedural knowledge of inquiry in the disciplines (Moje, 2015).

The idea of a hierarchical progression of disciplinary literacy, however, may be problematic, and is not accepted by all scholars. According to Rumelhart (1994), literacy growth is not a linear process, but relies on a variety of processors (i.e., syntactic, semantic, orthographic, and lexical) to converge simultaneously. Brozo et al. (2013) argue that the field needs to resist a “false dichotomy” (p. 354) between content area and disciplinary literacy practices. In resisting the dichotomous relationship, and “reconciling the divide” (Cervetti, 2014) content area literacy and disciplinary literacy should be viewed as complementary practices. In order to bridge content area reading with disciplinary literacy, some researchers have proposed discipline-specific strategies to aid students in constructing knowledge. Fisher, Grant, and Frey (2009) believe that modeling and reciprocal teaching give students a chance to practice, work, and communicate with others. Fang and Schleppegrell (2010) propose functional language analysis (FLA), which requires students to deconstruct how language is used differently across the disciplines. FLA gives students the tools necessary for close reading and engages them in doing, sensing, being, and saying in order to construct meaning from the text. Skills such as close reading, understanding complex vocabulary words and sentence structures, and an awareness of the author and context are a few of the skills necessary in disciplinary reading since this kind of reading is dictated by the text and moves from the inside out (Brozo et al., 2013). In “inside out” reading, the content area text governs the goals and processes needed for reading. Ultimately, content determines the process (Herber, 1970).

Previous research and literature have conceptualized disciplinary literacy in the four different content areas. However, we propose that the singular disciplinary literacy should be replaced with multiple disciplinary literacies. Talking about disciplinary literacies in the plural spotlights the variance dependent on discourse communities. As discussions among literacy theorists shift away from content-area literacy toward discussions of disciplinary-specific literacies (Shanahan & Shanahan, 2008; Draper, 2008), it is important to understand discipline-specific discourses. Even though this seems intuitive given current definitions of disciplinary literacy, the literacy field does not typically use the term disciplinary literacies. The plural offers not only the distinction among disciplines, but also the acknowledgment that there is a continuum of disciplinary literacy practices from novice to expert. There are, however, differing positions on this issue.

### Literacies within four disciplinary domains

We discuss four subject areas (i.e., ELA, science, history and social studies, and mathematics) and provide support from the literature about how each has its own specialized language, expectations, and means of constructing knowledge. In the context of secondary schools, the subject area of ELA is comprised of subdisciplines, such as literature, creative writing, and linguistics; science is comprised of subdisciplines, such as biology, physics, and chemistry; social studies is comprised of subdisciplines, such as history, economics, and psychology; and mathematics is comprised of subdisciplines, such as calculus, logic, and statistics. For ELA and social studies, we narrowed the focus to the subdisciplines of literature and history. We were able to find multiple studies that focused on literature specifically and history specifically at the secondary level, but we were not able to find a body of work centered on subdisciplines in math or science. For science and mathematics, we kept our focus more general on the subject areas. Our rationale is that the disciplinary literacy research on science asserts that scientific knowledge across subdisciplines develops in the same way by accruing “evidence for and against potential explanations of science phenomena” (Goldman et al., 2016, p. 12; Sandoval & Millwood, 2005). The same argument can be made for the subdisciplines in mathematics (Hillman, 2013).

We chose to focus on reading and writing like a literary critic, scientist, historian, and mathematician because these disciplinary identities have literature supporting differentiated discourses and related practices. We also anticipated that the study results could contribute to policy discussions, since these four disciplines are prominently represented in the Common Core State Standards for literacy and for mathematics.

#### Literacy in ELA

Almost by default, ELA is used as the model for literacy and the core of the disciplines because literacy skills are a central element of ELA. Specific to the field of ELA is the discipline of literary studies, with a tradition of expert-novice research on reading literature. This research offers insights into ways of reading and discourse practices of expert literary critics.

Texts serve as the primary objects of study for literary critics (Galloway et al., 2013), and reader-text relationships are multidimensional, formed around contextual, cognitive, and aesthetic dimensions. Integral to understanding a text is being able to situate the text in the historical, political, and cultural context in which it was written. Active engagement, or the cognitive dimension, includes the mental work necessary to interpret, analyze, and critique a text from a critical perspective. In order to fully understand a piece of literature, a literary critic constructs both literal and inferential understandings of a text through close reading (Reynolds & Rush, 2017). Close reading puts the text at the center of reading and requires readers to analyze the literal content (i.e., characterization, setting, and plot), the inferential (i.e., figurative language, diction, narration, and structure), and the interpretative (i.e., the reader’s personal experiences and literary theory) to make meaning (Hillocks & Ludlow, 1984; Lee, 2007). Close reading in ELA can be used to understand the author’s craft and decisions as well as develop an emotional response to the literature (Lee, 2007; Park, 2013; Rainey, 2017). This aesthetic response—or personal and emotional response—to literature is created through the reader’s dialogic transaction with the text and conversations with others (Rackley & Moyes, 2014; Reynolds & Rush, 2017; Rosenblatt, 1978). The aesthetic dimension engages students in using the text as a vehicle for “exploring the self and the world” (Park, 2013, p. 369), increasing readers’ empathy by helping them understand the human condition (Alsup, 2015; Grierson & Nokes, 2010), and gaining a deeper appreciation of the work (Rainey & Moje, 2012). In ELA discourse, the message about the world is called the theme.

The most important step in producing a persuasive and viable interpretation of a text is supplying evidence to support one’s claims, which can be drawn from three different sources: the self, the text, and literary criticism (Feldman, 1996; Rainey, 2017). According to Galloway et al. (2013), literary critics “assume a less detached stance” than scientists or historians as personal experience can be a source of evidence and emotional responses are important to the construction of arguments (p. 28). Literary critics also draw on knowledge of literary theory to inform interpretations and warrant claims (Goldman et al., 2016). Theories that literary experts draw upon include new historicism, reader response, feminist, queer, postcolonialism, critical race theory, and post-structuralism. Expert readers can construct various interpretations and value dialogue as part of the meaning making process (Feldman, 1996; Galloway et al., 2013; Goldman et al., 2016; Rainey, 2017; Reynolds & Rush, 2017). Thus, expert readers can construct deeper understandings of texts through placing the text within the historical context in which it was written, close analysis of the text’s content and author’s craft, and drawing on literary theory.

#### Literacy in science

Science literacy and disciplinary literacy in science can mean two different things within the field. Some use *science literacy* to refer to understanding the nature of science and knowledge about scientific processes (Norris & Phillips, 2003), while disciplinary literacy means competency in reading, writing, and thinking processes in science. In science, reading and writing are tools (Cervetti & Pearson, 2014) used by scientists to systematize, describe, and explain natural and designed worlds (Goldman et al., 2016; Good, Shymansky, & Yore, 1999). Alongside firsthand experiments, reading texts provides secondhand experiences to help scientists build background knowledge and discover scientific consensus on a topic. Scientists write arguments to construct claims, inform, and persuade others to act on scientific problems (Yore, Bisanz, & Hand, 2003).

Science has its own style, genres, and discourse conventions that both overlap with and differ from other disciplines (Halliday & Martin, 1993). Scientists write in a clear style to record and inform (Day, 1992) and align claims and evidences to persuade (Holland, Holyoak, Nisbett, & Thagard, 1986). Fang (2013) lists the major genres of academic science as, “procedure, procedural recount, description, report, explanation, and exposition” (p. 277). After a synthesis of the literature, three key discourse conventions of science literacy became clear. Becoming literate in science requires (a) knowledge of scientific terminology, (b) synthesis of multiple sources, and (c) analytical thinking.

First, one must learn the vocabulary and style of science to be able to read and write like a scientist. Scientists create words based on Latin morphemes and nominalization structures (Day, 1992; Fisher et al., 2009; Shanahan et al., 2011). Also, scientists use words like “suggest” and “predict” to reveal their level of certainty. Readers in science pay attention to these stylistic clues to interpret the author’s findings (Goldman et al., 2016; Norris & Phillips, 2003). Second, scientists are required to synthesize information presented in multiple forms within a text and synthesize information across texts. Scientists interpret prose as well as pictures, charts, diagrams, equations, and tables (Goldman et al., 2016; Hand et al., 2003; Shanahan & Shanahan, 2008). In addition to synthesizing multiple texts when reading, scientists do so when writing, including representing data using multimedia (Yore et al., 2003). The third key convention is that science and analysis go hand in hand (Yore et al., 2003). Scientists link data directly to the inferences and conclusions made in an argument (Gillis, 2014).

Evaluation of quality is different in science than the other disciplines (Norris & Phillips, 2003; Shanahan et al., 2011). When scientists choose a text, they may evaluate based on the author’s credentials, but when they read, they analyze the rigor of the procedures outlined and the plausibility of the argument rather than analyze the author’s point of view (Shanahan et al., 2011). Scientists evaluate procedures, analysis, and claims based on completeness and consistency in tandem (Bricker & Bell, 2008; Norris & Phillips, 2003). Understanding these genres, discourse conventions, and measures of quality is essential for disciplinary literacy in science.

#### Literacy in history and social studies

Research on disciplinary literacy in social studies has emerged over the last few decades in three phases: (1) the situating of literacy in the context of disciplinary knowledge, (2) the emergence of a cognitive argument for disciplinary literacies, and (3) the rise of pedagogical scaffolds for supporting literacy teaching and learning. Concurrent with educational reform and the publication of *A Nation at Risk* in 1983, educators began to push for new ways to conceive of literacy in history and social studies as rooted in disciplinary ways of knowing. Supporting this view, Gagnon (1989) described disciplinary literacies as shaped by an understanding of historical significance, historical causation, and the ability to “recognize the difference between fact and conjecture, between evidence and assertion, and thereby to frame useful questions” (Gagnon & The Bradley Commission on History in Schools, 1989, pp. 25–26). Historians and history educators have expanded upon this notion of literacy as uniquely situated in disciplinary knowledge to include, more generally, the capacity to construct arguments and to use evidence effectively (Bain, 2005; Holt, 1990; Leinhardt, Beck, & Stainton, 1994; VanSledright, 2002).

Extending the idea of literacy as disciplinary knowledge, scholars have increasingly sought to identify domain-specific cognitive skills that are unique to history (Lévesque, 2008; Monte-Sano, 2010, 2011; Thornton & Barton, 2010). Wineburg’s groundbreaking research (1991a, b) examined the differences in how historians and students approached reading historical texts. His findings included not only different ways of reading but different ways of constructing knowledge. While reading historical documents, historians viewed the practice of reading as part of investigation and considered human motives, degree of trustworthiness, and author purpose (Wineburg, 1991b). Building on the Wineburg’s three-part heuristic for reading and analyzing historical sources, which includes summarization, contextualization, and corroboration, the Stanford History Education Group has led the way in creating curriculum materials for scaffolding historical thinking. The *Reading Like a Historian* curriculum of document-based lesson plans is premised on research which suggests that disciplines—in this case, history—have particular modes of thinking that cannot be assimilated as general skills and thus, require teaching materials designed narrowly to produce thinking and literacy skills within the discipline (Reisman, 2012). Reisman’s (2012) experimental study found students who received the disciplinary literacy curriculum performed better on the outcome measures of historical thinking, transfer of historical thinking, factual knowledge, and reading comprehension. Other expert-novice research in the discipline of history include Beck and McKeown (2002), who found that adult readers used the strategy of questioning the author to infer implied meanings behind the text while students needed more scaffolding. Leinhardt and Young’s (1996) study found that historians engaged in expert reading through classification, sourcing, corroboration, and contextualization. Historians also engaged in reading documents as text, as artifacts, and as part of a set of related texts by examining text structures and word choice while also interpreting the meaning of the document through historical theory.

#### Literacy in mathematics

In a recent review, Hillman (2013) introduces discourse as a key feature of mathematical literacy, which encourages a broad view of how mathematicians communicate. Arguing that discourse and communication have long been at the heart of mathematics (see Yore, Pimm, & Tuan, 2007; O’Halloran, 2005), Hillman claims that mathematical literacy requires students to explain their reasoning. The emphasis on communication and mathematics is prominently displayed in the third CCSS standard, which states that students should learn to construct arguments and critique those of others.

Within mathematical instruction, emphasis is placed on the reasoning process rather than uniquely on a correct answer since often students can learn to acquire the correct answer without understanding essential concepts well. Additionally, mathematicians must “connect to other problems that can be solved the same way, identify patterns, read and represent findings visually, know definitions of words and symbols, think abstractly, and verify their answers” (Hillman, 2013, p. 398). Reading a mathematics text is complex because it includes dense language, numeric symbols that need to be decoded, graphics, as well as a lack of redundancy (Metsisto, 2005; Schleppegrell, 2007). Teaching students to be quantitatively literate requires them to think mathematically and apply concepts, which is different from teaching them mathematical content (Piatek-Jimenez, Marcinek, Phelps, & Dias, 2012). Guiding students to think like mathematicians requires them to speak using the language of the discipline, which plausibly leads to a deeper understanding of mathematical concepts. Fang (2012) further elaborates on the role of discursive features in mathematics by claiming that language, symbolism, and visuals “interact in synergistic ways to construe mathematical knowledge, processes and reasoning” (p. 53). Expertise in meaning-making resources promotes student success with mathematical discourse.

Research on the literacy skills distinct to mathematics emerged in the late 1980s. Tall’s (1991) *Advanced Mathematical Thinking* examined empirical studies involving both mathematicians and students. Thurston (1990) asserted that the mental representation of the mathematical reasoning process is a compression of the complex foundational concepts. Taking this into account when writing, mathematicians have to explicate the reasoning and when reading, mathematicians have to construct the reasoning behind the problem solving if it is not made clear by the author (Siegel & Fonzi, 1995). Readers visualize problems to create mathematical modeling and then create mathematical representations. Quantitative literacy, spatial literacy skills, and mathematical communication relate mathematical representations to models and solve real world problems (NCTM, 1989, 2000).

### The current study

In order to operationalize disciplinary literacy, we focused on literacies associated with the four subject areas of ELA, science, history and social studies, and mathematics. As previously mentioned, we concentrated on these four areas because they are well developed, with distinct bodies of literature. Additionally, since the CCSS focuses on these core areas, providing insights about related construct validity provides foundational evidence that supports the decision to emphasize literacy within these disciplines.

Our goal was to target the key literacy practices that teachers in each discipline engage in during reading and writing within their disciplines. Our approach to target these practices was twofold. First, we reviewed the professional literature in each of the four disciplines to reach an understanding of what practices are deemed particular to that discipline. Second, we conducted focus groups with teachers in each discipline to confirm the practices that were noted in the literature. We then generated survey items based on the consensus of key practices within each discipline.

Next, we empirically tested the relationship among those practices with teachers from the four disciplines. A self-report survey was developed for measuring teachers’ practices regarding emerging constructs of disciplinary literacies within four core subject areas. The goal was to ascertain the current status of teachers’ self-reported practices within the four subject areas for instructors who teach in grades 6-12. We posed two research questions: (a) What are teachers’ self-reported disciplinary literacy practices in these four areas?; and (b) Do disciplinary literacies in fact differ within these four areas?

## Methods

In this section, the procedures and methods used to develop, explore, confirm, and test a scale of disciplinary literacy are outlined. First, item development, content validation, and gathering of participants are discussed, followed by the method used to remove careless responders. Next, the cleaned dataset demographics are outlined, followed by a description of the exploratory factor analysis, preliminary factor structure, and method used for item retention. Finally, the procedures that were used to confirm the factor structure and test differences among disciplines are described.

### Survey development

The online survey was deployed through Qualtrics with the survey development process consisting of scale development, item creation, content validation, and choice of response format.

#### Item development

Our first task was to define the construct of disciplinary literacy through synthesis of relevant literature and expert judgment (Hinkin, 1998; Spector, 1992). To guide scale development, disciplinary literacy was defined as “the use of reading, reasoning, investigating, speaking, and writing required to learn and form complex content knowledge appropriate to a particular discipline” (McConachie & Petrosky, 2010, p. 6). This working definition was chosen because it represented a comprehensive perspective on disciplinary literacy that served the dual function of targeting key literacy processes and acknowledging discipline particularity. In order to operationalize the particularity of disciplines we synthesized the disciplinary literacy literature within four core areas as mentioned earlier in our theoretical and conceptual section. Next, content experts from the four disciplines (i.e., professors, doctoral students, and master teachers from ELA, science, history and social studies, and mathematics) gathered to write items based on the agreed upon definition. A collaborative session of item writing produced over 100 items. The large bank of items was generated so as to adequately sample from the true construct domain as suggested by Hinkin (1998).

#### Content validation

The content experts rated each item within the 100-plus-item pool for content relevance and item quality. Content experts rated each item as essential, useful but not essential, or not necessary. A content validity ratio (CVR) was then calculated for each item, providing a structured method to assess content validity (Lawshe, 1975). Items with high content validity and quality were retained. Items with essential content but low quality were revised for quality, and the remaining items were screened for duplicates. Finally, the wording of the items were revised for consistency and then screened through cognitive interviews (Groves et al., 2009) with two focus groups of middle school teachers (*n* = 8) and two focus groups of high school teachers (*n* = 8). The cognitive interview findings were used to clarified procedures, item wording, and response choices. This process resulted in 24 finished items.

#### Response format

Frequency scale anchors ask the individual to recall and cognitively compute how often a specific primed behavior has been engaged in (Schwarz & Oyserman, 2001). The mental process of responding to a frequency scale focuses attention on how often an act occurs. Agreement scales on the other hand focus participants’ attention on the propensity to engage in a behavior and may actually assess attitude toward a behavior rather than prior experience of that behavior (Dalal, 2005). Since disciplinary literacy was defined as “the use of reading, reasoning, investigating, speaking, and writing” (McConachie & Petrosky, 2010, p. 6), past patterns of individuals’ actual behavior were of interest. As such, items were anchored on a five-point Likert frequency scale that ranged from always to never. A five-point format is considered best practice for Likert scales, with minimal increase in statistical difference associated with more points on the scale (Hinkin, 1998; Morata-Ramírez & Holgado-Tello, 2013). Items were grouped in clusters with sentence stems asking participants to rate the frequency of reading or writing in the discipline in which they taught. For example, if a teacher selected science as her subject area, the sentence stem would state: When reading in science, how often do you analyze quantitative terminology? The five-point options included never, seldom, sometimes, often, always.

### Sample and survey administration

Participants were solicited via both purposeful and snowball sampling techniques (Bogdan & Biklen, 1998). The sample was recruited through advertisements on listservs of professional networks (i.e., LRA, NCSS, NCTE, NCSS) and personal networks using social media. Participants in the network were asked to forward the web survey to sixth—twelfth grade teachers in ELA, science, history and social studies, and mathematics. The survey was open for 30 days.

**Fig. 1:**
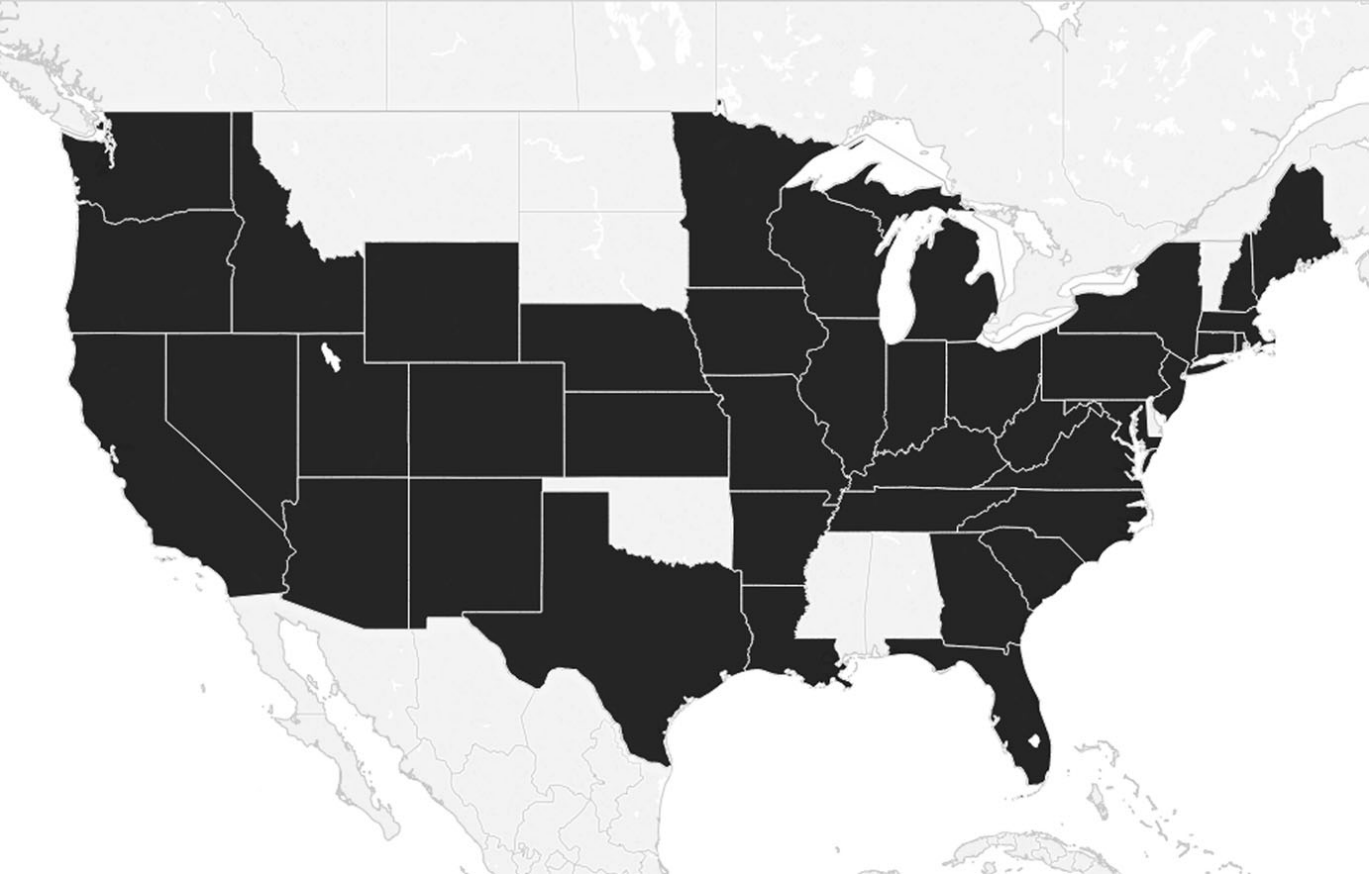
States where participants responded

Participants comprised sixth—twelfth grade teachers who were teaching ELA, science, history and social studies, or mathematics. Experts can be defined as researchers of the construct or members of the target population who have direct experience with the construct (Vogt, King, & King, 2004). We chose to survey sixth—twelfth grade teachers, as our study was specific to disciplinary literacy in the context of secondary schooling. We realize that establishing teachers as disciplinary experts is a departure from early studies such as those conducted by Shanahan and Shanahan (2008) and others, and consider this choice of participants as a key feature of our study. We agree with Goldman et al. (2016) and Moje (2010) who position secondary teachers as practitioners of their disciplines. Table 1 provides demographics of participants. US averages were retrieved from Feistritzer’s (2011) national sample. A total of 857 people completed the survey. Participants from 41 states were represented in the usable data set of 820 respondents (ELA = 320, science = 229, history and social studies = 161, mathematics = 110). See Fig. 1 for graphic distribution of the sample by state.

**Table 1:** Profile of Participants

Professional experience and demographics	Number of respondents	Percentage of respondents	US average^a^
Grade level taught			
6–8	376	47.8	
9–12	411	52.2	
Years of teaching experience			
0–5	144	18.2	26
6–10	209	26.5	16
11–15	159	20	16
16–20	116	14.7	23
21–25	67	8.5	
26 or more	94	11.9	17
Highest degree attained			
Bachelor	273	34.7	44
Graduate	513	65.3	56
Licensed in teaching area			
Licensed	762	96.6	
Not licensed	27	3.4	

^a^From Feistritzer (2011)

### Preliminary data analysis

The distributed surveys resulted in 857 completed surveys. As careless responding is a problem with Internet survey research, prior to analysis the datasets should be screened for careless responding with at least one method (Meade & Craig, 2012). The longest string method was used to compute an indicator of careless responding. The longest string is the maximum number of consecutive items with the same response choice for each respondent across the 24 scale items. A cutoff value for the maximum longest string was formed based on the clear breakpoint in the frequency distribution (Johnson, 2005). The long string flag value was > 10, and participants with longer strings were excluded for careless responding (4%). Following best practices (Fabrigar, Wegener, MacCallum, & Straughan, 1999) the resulting 820 participants were randomly split in half, 410 for the exploratory factor analysis and 410 for a confirmatory factor analysis.

### Exploratory factor analysis

All analyses and data transformations were performed with the open source software R. The first sample of observations (*n* = 410) was assessed through exploratory factor analysis (EFA) on Pearson correlations. Principal axis factoring with direct oblimin oblique rotation was utilized to account for the possible correlation of latent variables, and estimated with maximum likelihood conducted in the *psych* package in R (Fabrigar et al., 1999; Revelle, 2014).

Prior to the EFA, the Kaiser–Meyer–Olkin (KMO) measure was utilized to test sampling adequacy of items to yield distinct and reliable factors (Field & Zoe, 2012). The KMO values indicate that the sample adequacy was good (KMO = 0.86.), even “meritorious” according to Hutcheson and Sofroniou (1999). The KMO value for each item was greater than 0.75 except for one item, which was eliminated from subsequent analyses.

To determine the most adequate number of factors to extract, initially a scree plot was used (Cattell, 1966). No decisive solution was evident, as the scree showed many interpretable possibilities with 2, 3, 4, or 6 extracted factors. To reach a more conclusive and empirical decision, Veckler’s MAP method and very simple structure (VSS) were used, both converging on a 3 factor solution. Veckler’s MAP is a useful metric as it rewards parsimony, rejecting components identified by only one variable, and is more accurate and stable than the Kaiser test, Barlett, or scree methods (Zwick & Velicer, 1986). The choice of a 3-factor extraction was strengthened by the convergence of the VSS likewise recommending the extraction of 3. Veckler’s MAP achieved a minimum of 0.02 with 3 factors and VSS complexity achieved a maximum with 3 factors with 0.81, thus a three-factor solution was used in the EFA (see Table 2 for results).

**Table 2:** Individual item loadings for the 3 factor EFA

	Disciplinary literacy items	Factor I	Factor II	Factor III	Keep
		Source literacy	Analytic literacy	Expressive literacy	
1.	When writing, how often do you revise for credibility of other authors’ claims?	**0.79**	0.00	0.04	●
2.	When evaluating author’s claims, how often do you verify the author’s claims with other authors’ claims?	**0.74**	− 0.04	0.05	●
3.	When synthesizing information, how often do you require corroborating evidences from multiple authors?	**0.69**	0.04	− 0.02	●
4.	When evaluating author’s claims, how often do you consider the author’s institutional affiliation?	**0.62**	− 0.07	− 0.04	●
5.	When writing, how often do you revise for validity and replicability?	**0.53**	0.32	− 0.02	●
6.	While reading, how often do you analyze quantitative terminology?	− 0.02	**0.72**	0.11	●
7.	While reading, how often do you analyze technical terminology?	− 0.02	**0.67**	0.13	●
8.	When reading how often do you analyze graphs?	0.05	**0.63**	− 0.29	●
9.	When synthesizing information, how often do you interpret data to support a model or hypothesis?	0.23	**0.52**	− 0.11	●
10.	How often do you organize curricular material by escalating logic?	− 0.05	**0.49**	0.01	●
11.	When synthesizing information, how often do you aim for convergence upon a solution?	0.11	**0.47**	0.01	●
12.	How often do you organize curricular material by progression of topics?	− 0.07	**0.46**	− 0.03	●
13.	While reading, how often do you deconstruct figurative language?	− 0.11	0.09	**0.85**	●
14.	When reading, how often do you analyze a text’s rhetorical devices?	0.12	0.00	**0.69**	●
15.	When evaluating author’s claims, how often do you differentiate the speaker from the author?	0.31	− 0.18	**0.60**	●
16.	When writing, how often do you revise for style and voice?	0.39	− 0.11	**0.46**	●
17.	While reading, how often do you analyze source information?	0.29	0.21	0.37	○
18.	How often do you organize curricular material by themes?	0.14	0.12	0.20	○
19.	How often do you organize curricular material by chronological progression?	0.01	0.17	0.20	○
20.	When reading, how often do you analyze photographs as source information?	0.37	0.21	0.10	○
21.	When writing, how often do you revise for precision and accuracy?	0.44	0.26	0.08	○
22.	When reading, how often do you analyze symbolic notation?	0.02	0.41	0.21	○
23.	When evaluating author’s claims, how often do you consider information about the author inconsequential?	− 0.02	0.10	− 0.02	○
24.	When synthesizing information, how often do you include personal experiences as evidence?	0.01	0.15	0.26	○

### Retaining items

For item elimination, Comrey and Lee’s (1992) criteria of fair loadings was used, which states that primary factor loadings above 0.63 are very good, above 0.55 are good, and above 0.45 are fair. All items that passed as fair (loadings above 0.45) were retained at this point in the analysis. This resulted in a 16-item disciplinary literacy scale, as represented by the bold loadings in Table 2. The retained items showed good factor structure with minimal cross loadings on secondary factors. The three-factor solution using retained items explained 49% of the variance. Under some circumstances, it is desirable to account for more than 50% of the variance explained through factor structure alone (Marsh, Hau & Wen, 2004). In this particular case it is important to note that the three-factor structure accounted for practically 50% of the variance before parceling out unique discipline effects and was the best fit given the data. Arguably, disciplinary literacy should differ theoretically across disciplines (as this study was seeking to investigate). Accordingly, when controlling for discipline, the variance accounted for by factor structure increased, as described in subsequent analyses.

The three-factor solution was interpreted as the first factor representing *source literacy*, the second as *analytic literacy*, and the third factor as *expressive literacy*. These three terms were chosen because they represent the factor in a comprehensive way. Source literacy, which is most directly related to the literature introduced by Wineburg (1991a, b), is concerned with authorship in order to gain credibility as a reader and writer. The term analytic literacy, which we derived from the literature, focuses on solution-oriented thinking, quantitative and technical terminology, as well as visual representations such as graphs and models (for a review, see Hillman, 2013; Norris & Phillips, 2003). Expressive literacy is conceptually grounded in the broad field of literary criticism, specifically associated with the new critics (e.g., Richards, 1929). Expressive literacy involves deconstructing and generating literary devices (e.g., figurative language, rhetoric, and narration) with attention to voice and style.

### Confirmatory factor analysis methods

The method of confirming the factor structure followed best practices in theory development and was accomplished with the random split-half method (Fabrigar et al., 1999). To test the factor structure, the second half of the split-half data was analyzed with confirmatory factor analysis (CFA), conducted on the 16 retained items in three correlated latent three factors using maximum likelihood estimation. The three-factor model assumed that the items were driven by three latent literacy factors: (a) source literacy, (b) analytic literacy, and (c) expressive literacy. Model fit was evaluated using the comparative fit index (CFI; Bentler, 1990), Standardized Root-Mean Square Residual (SRMR, Bentler, 1995), and the root mean square error of approximation (RMSEA; Browne, & Cudeck, 1993). SRMR indicates a better fit when small: 0 equaling perfect fit, values below 0.8 indicating good fit, and values below 0.10 considered acceptable (Hu & Bentler, 1999). RMSEA indicate moderate fit when below 0.8 (MacCallum, Browne, & Sugawara, 1996). CFI values range from 0 to 1, with values roughly above 0.9 indicating a good fit (Marsh et al., 2004).

## ANOVA testing for differences among subject areas

One-way ANOVAs were applied to the data to test mean differences among the different four subject areas. ANOVAs allow testing of these differences only if assumptions of homogeneity of variance between groups are met. These assumptions of homogeneity were evaluated with Levene’s test. Running Levene’s test revealed significant deviations from homogeneity between subject areas by analytic literacy *F*(3, 816) = 3.57, *p* < 0.001, source literacy *F*(3, 816) = 5.87, *p* < 0.001, and expressive literacy *F*(3, 816) = 12.83, *p* < 0.001. Since the assumption of homogeneity of variance was not met, Welch’s adjusted F-test was used to assess mean differences. For effect size measures, adjusted omega squared (ω^2^) values were calculated based on the F-tests. For post-hoc comparisons of literacy by subject areas, standard box-plots were conducted as well as Dunnett’s T3 procedure (1980). Dunnett’s T3 produces slightly wider, more conservative confidence intervals than the Games-Howell procedure, both of which are standard analyses for multiple comparisons with unequal sample sizes and heterogeneous variances (Dunnett, 1980; Wilcox, 1987).

## Results

In this section we address both research questions by presenting findings based on the results of the confirmatory factor analysis and ANOVA of differences by each of the four core disciplines (i.e., ELA, science, history and social studies, and mathematics).

### Confirmatory factor analysis

Confirmatory factor analysis (CFA) was conducted with the second half of the randomly split original 820 cases to test the factor structure of the exploratory results. The proposed three-factor model originally revealed a marginal fit to the data (*X*^2^ (90, *n* = 410) = 295.36, *p* < 0.001, CFI = 0.92, SRMR = 0.07, RMSEA = 0.09). In efforts to improve fit, two items from analytical literacy and one from source literacy were removed due to low factor loadings (≤ 0.45) and weak alignment to theory (Brown & Moore, 2015; Comrey & Lee, 1992). These three questions asked the following:

- How often do you organize curricular material by escalating logic?
- How often do you organize curricular material by progression of topics?
- When writing, how often do you revise for validity and replicability?

**Fig. 2:**
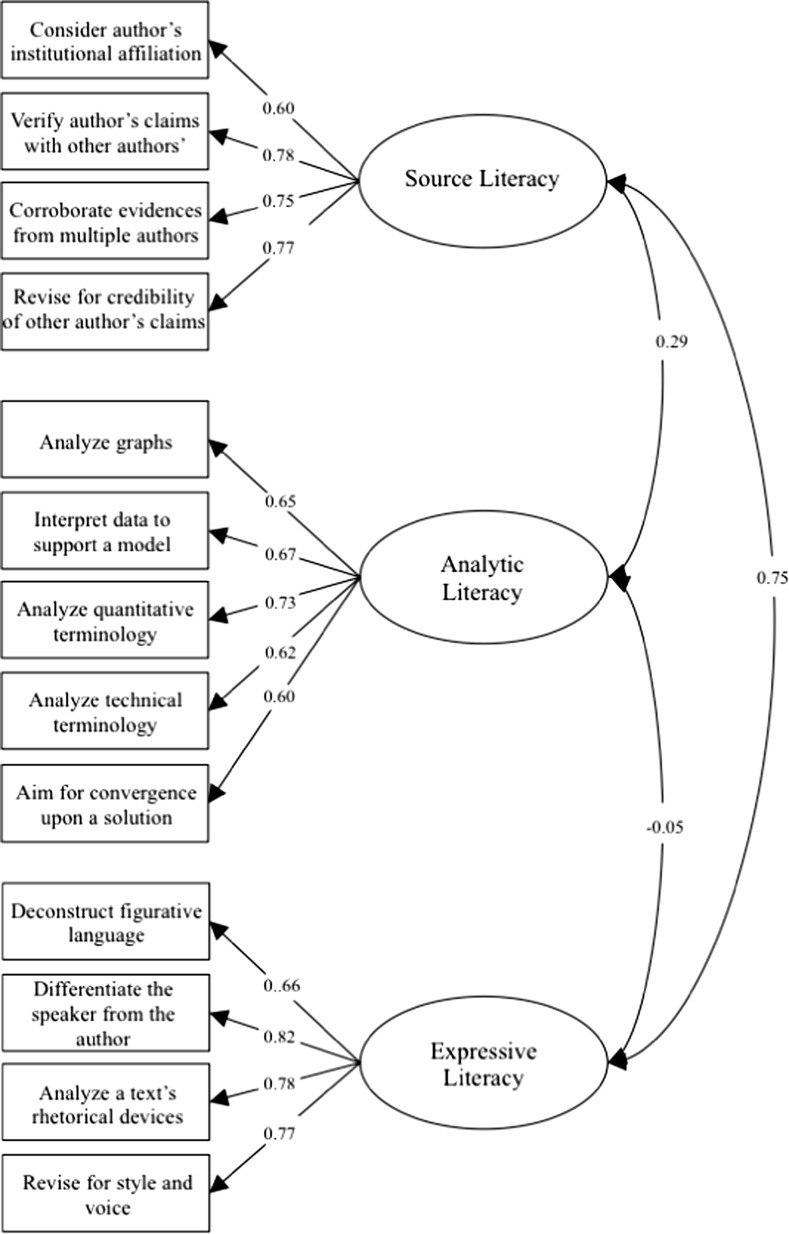
Standardized factor loading for the three-factor CFA

The first two items described instructional practices more than disciplinary literacy practices and were unlike the other items. Removing these three items resulted in a better fit (∆*X*^2^ (39) = 190.48, *p* < 0.001) and the 13 remaining items all displayed strong factor loadings (≥ 0.54). Overall, the revised model fit well (*X*^2^ (51, *n* = 410) = 104.88, *p* < 0.001, CFI = 0.98, SRMR = 0.06, RMSEA = 0.05) with acceptable CFI, SRMR, and RMSEA values. The retained model is presented in Fig. 2.

### Internal consistency

Reliability for each factor was estimated using Cronbach’s alpha. All reliabilities were adequate, with the source literacy internal consistency coefficient at 0.81, analytic literacy factor coefficient at 0.77, and the expressive literacy factor at 0.84 (see Table 3).

**Table 3:** Between-group differences for factors of disciplinary literacy

Factor	Cronbach’s *α*	ELA teachers	History teachers	Math teachers	Science teachers	*Df*	*Welch’s F*	ω^2^
		*n* = 320	*n* = 161	*n* = 110	*n* = 229			
		*M* (*SD*)	*M* (*SD*)	*M* (*SD*)	*M* (*SD*)			
Source literacy	0.81	3.30 (0.69)	3.48 (0.68)	2.10 (0.91)	3.37 (0.85)	3; 336.90	65.52***	0.24
Analytic literacy	0.77	3.18 (0.68)	3.54 (0.62)	3.93 (0.56)	3.88 (0.55)	3; 357.51	72.22***	0.21
Expressive literacy	0.84	4.02 (0.59)	3.20 (0.77)	2.29 (0.83)	2.81 (0.84)	3; 324.42	222.47***	0.43

*p < 0.05; **p < 0.01; ***p < 0.001

### Analysis of variance among subject areas

To test differences by subject areas, one-way ANOVAs with Welch’s adjusted F-ratio were conducted for each factor of literacy. Results indicated that the subject areas accounted for significant variance among the three factors of literacy (see Table 3). There was a significant effect of subject areas on source literacy, *Welch’s F* (3, 336.90) = 65.52, *p* < 0.001, *est.* ω^2^ = 0.24, as well as analytic literacy *Welch’s F* (3, 357.51) = 72.22, *p* < 0.001, *est.* ω^2^ = 0.21, and expressive literacy *Welch’s F* (3, 324.42) = 222.47, *p* < 0.001, *est.* ω^2^ = 0.43. The effect of subject areas on the three literacies all passed Cohen’s (1969) cutoff of 0.1379 as a large effect, with subject areas explaining between 21 and 43% of the variance in the three literacies.

### Post-hoc comparisons

In order to further assess the differences in disciplinary literacy practices, Dunnett’s (1980) modified Tukey–Kramer tests were conducted for each contrast by subject areas. Table 4 provides a full summary of post-hoc comparisons.

**Table 4:** Post-hoc analysis of mean differences by teaching area

Factor	Differences	95% Confidence intervals
Source literacy		
History versus ELA	0.18	[− 0.016, 0.366]
Math versus ELA	− 1.21***	[− 1.424, − 0.987]
Science versus ELA	− 0.04	[− 0.206, 0.137]
Math versus History	− 1.38***	[− 1.625, − 1.136]
Science versus History	− 0.21*	[− 0.413, 0.007]
Science versus Math	1.17***	[0.942, 1.400]
Analytic literacy		
ELA versus Math	− 0.74***	[− 0.920, − 0.567]
ELA versus Science	− 0.70***	[− 0.837, − 0.560]
ELA versus History	− 0.39***	[− 0.514, − 0.206]
Math versus Science	0.05	[− 0.140, 0.230]
Math versus History	0.38***	[0.186, 0.581]
Science versus History	0.34***	[0.175, 0.503]
Expressive literacy		
ELA versus Math	1.73***	[1.524, 1.942]
ELA versus Science	1.22***	[1.052, 1.379]
ELA versus History	0.83***	[0.644, 1.010]
Math versus Science	− 0.52***	[− 0.738, − 0.299]
Math versus History	− 0.91***	[− 1.140, − 0.672]
Science versus History	− 0.39***	[− 0.583, − 0.194]

*p < 0.05; **p < 0.01; ***p < 0.001

^a^All differences and confidence intervals were conducted with the Dunnett (1980) modified Tukey–Kramer test

#### Source literacy

**Fig. 3:**
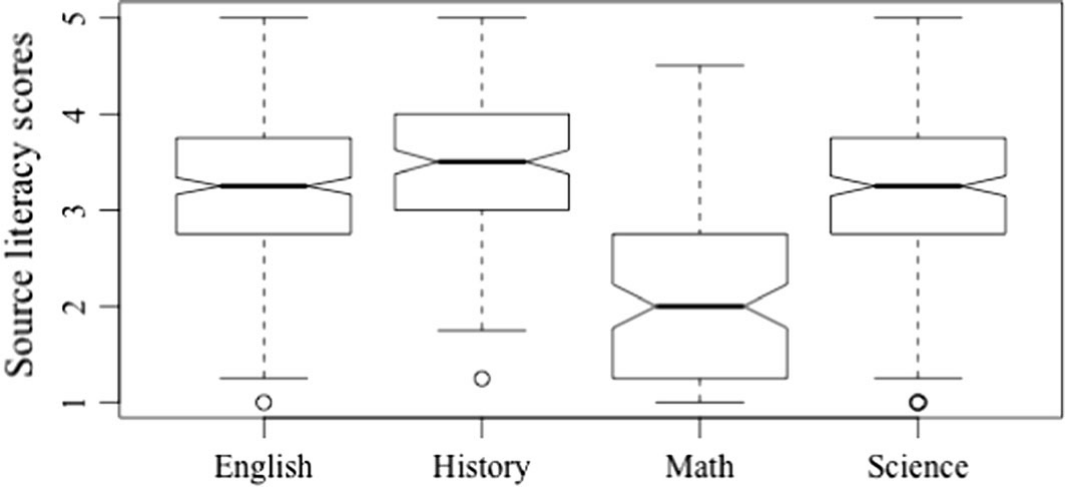
Source literacy by teaching area

Teachers differed significantly with regard to their practice of source literacy (*Welch’s F* (3, 336.90) = 65.52, *p* < 0.001, *est.* ω^2^ = 0.24), which is graphically represented in Fig. 3. Specifically, teachers of mathematics engaged in significantly less source literacy practices than teachers of ELA (T3 = − 1.21, *p* < 0.001), science (T3 = − 1.17, *p* < 0.001), and history and social studies (T3 = − 1.38, *p* < 0.001). Teachers of history and social studies were the highest in source literacy (*M* = 3.48), followed closely by those who taught ELA (*M* = 3.30). Teachers of history and social studies scored significantly higher in source literacy than those who taught science (T3 = 0.21, *p* < 0.05) and those who taught mathematics (T3 = 1.38, *p* < 0.001). Teachers of science did not significantly differ from those who taught ELA (T3 = − 0.04, *p* > 0.05) with respect to source literacy.

#### Analytic literacy

**Fig. 4:**
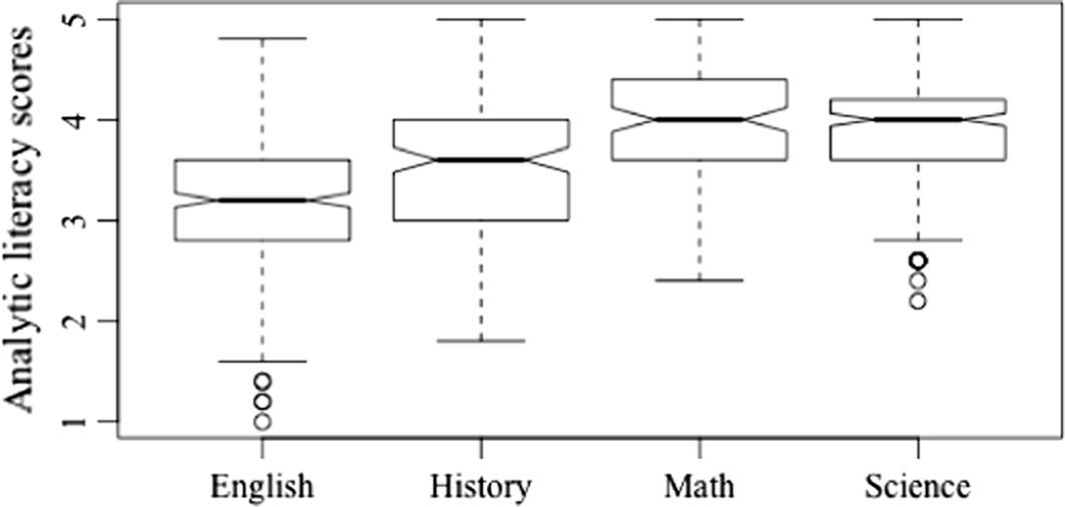
Analytic literacy by teaching area

The practice of analytic literacy also differed significantly across subject areas (*Welch’s F* (3, 357.51) = 72.22, *p* < 0.001, *est.* ω^2^ = 0.21). Figure 4 provides a graphic representation. Teachers of mathematics engaged in the highest levels of analytic literacy (*M* = 3.93), with significantly higher reported levels than teachers of ELA (T3 = 0.74, *p* < 0.001) and history and social studies (T3 = 0.38, *p* < 0.001). Teachers of ELA reported the lowest levels of analytic literacy, less than those in science (T3 = − 0.70, *p* < 0.001), history and social studies (T3 = − 0.39, *p* < 0.001), and mathematics (T3 = − 0.74, *p* < 0.001). Teachers of science did not significantly differ in their use of analytic literacy from those who taught mathematics (T3 = − 0.05, *p* > 0.05), but both were higher in their use than those who taught ELA.

#### Expressive literacy

**Fig. 5:**
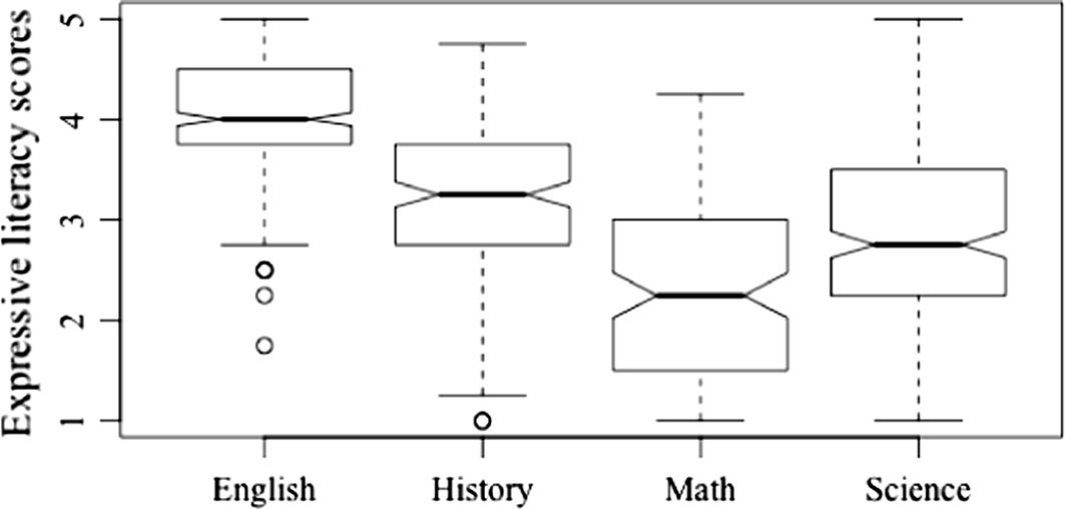
Expressive literacy by teaching area

Post-hoc comparisons of expressive literacy revealed significant differences between all four subject areas (*Welch’s F* (3, 324.42) = 222.47, *p* < 0.001, *est.* ω^2^ = 0.43). Figure 5 provides a graphic representation of differences. Teachers of ELA engaged in the highest levels of expressive literacy (*M* = 4.02), higher than teachers of mathematics (T3 = 1.73, *p* < 0.001), history and social studies (T3 = 0.83 *p* < 0.001), and science (T3 = 1.22, *p* < 0.001). Teachers of mathematics engaged in the lowest levels of expressive literacy, with significantly lower levels of expressive literacy than teachers of ELA (T3 = − 1.73, *p* < 0.001), science (T3 = − 0.52, *p* < 0.001), and history and social studies (T3 = − 0.91, *p* < 0.001). Lastly, teachers of history and social studies engaged in significantly more expressive literacy than teachers of science (T3 = 0.39, *p* < 0.001).

## Discussion

The research attempted to (a) define disciplinary literacies within four subject areas (ELA, science, history and social studies, and mathematics); (b) assess teachers’ self-reported practices with respect to disciplinary literacies in these four core areas; and (c) empirically demonstrate how disciplinary literacies differ within these four areas. In the following discussion, we summarize and interpret the findings and relate the findings to existing research on disciplinary literacy.

### Summary and interpretation of findings

The primary interest of this study was to investigate the construct of disciplinary literacy as teacher practice, operationalized through the areas of ELA, science, history and social studies, and mathematics. The findings supported the notion that disciplinary literacy is, in fact, a series of at least three *disciplinary literacies*: source literacy, analytic literacy, and expressive literacy. After confirming this three-factor structure via CFA, differences by literacy and discipline were evaluated. Findings supported individual-level differences in which teachers engaged in some amount of each of the three factors. When teachers were grouped by subject area taught, patterns emerged suggesting that these disciplinary-specific teachers were more likely to engage in certain literacies. Results indicated that literacy practices are not limited to the ELA classroom, but instead are distributed among teachers of academic subjects with source, analytic, and expressive literacies practiced by varying degrees.

Source literacy includes the reading skills of sourcing (e.g., determining an author’s point of view), contextualization (e.g., determining what was going on when the text was produced), and corroboration (e.g., finding other versions of a story supporting the ideas within; Wineburg, 1991b). History and social studies teachers reported engaging the most with source literacy. For example, when reading and writing, history and social studies teachers encourage students to carefully consider the credibility of claims they are using from text. When evaluating those author’s claims, history and social studies teachers also encourage students to verify the author’s claims with other authors’ claims as well as requiring corroborating evidences from multiple authors. Based on this finding, history and social studies teachers are positioned as experts to teach source literacy practices to their students.

Analytic literacy involves quantitative reasoning and deconstructing technical terminology, graphs, and models. Mathematics and science teachers reported engaging the most with analytic literacy. For example, when reading through an analytic literacy lens, readers must move between prose and visual representations to understand the author’s claim and interpret data to support models. Additionally, mathematics and science teachers tend to aim for convergence when reading and writing to support a conclusion. Ideally, mathematics and science teachers, as experts on analytic literacy, would include teaching their domain-specific literacy to their students.

Expressive literacy includes deconstructing figurative language and rhetorical devices. For example, readers interpret layers of meaning to understand literal meaning and infer figurative meanings. A reader looks at author’s craft including differentiating from the speaker and the author; writers revise for style and voice. In the survey, ELA teachers reported engaging the most with expressive literacy. ELA teachers are the experts on expressive literacy, but may not be experts in the other two literacies (source and analytic).

The idea of literacy as a distributed practice that emerged in this research aligns with the idea of a shared responsibility for literacy instruction as encouraged in state and national standards for ELA (e.g., NGA Center & CCSSO, 2010). The findings of this research suggest that multiple types of disciplinary literacies exist and as such are best facilitated by academic teachers within the context of the disciplines in which they are most heavily practiced. This notion of disciplinary literacies aligns well with the CCSS insistence that “college and career ready students be proficient in reading complex informational text independently in a variety of content areas” (NGA Center & CCSSO, 2010, p. 4).

What surprised us about the findings was that science was not clearly represented by a discrete factor. In fact, science teachers used both analytic and source literacies. Specifically in science, experts corroborate findings with previous research; however, science experts also conduct analytical readings in ways similar to mathematicians. Perhaps if we had separated sciences into life sciences, physical, and earth and space, we would have derived different correlations among the different sub-fields. For example, chemistry and physics may have aligned more with analytic literacy because of the high use of symbolic notation, while life sciences potentially could have aligned more with source literacy because of the high reliance on corroboration with other studies.

Based on our results from respondents, we can conclude that there are at least three types of literacies in operation among the four core disciplines of ELA, science, history and social studies, and mathematics. This is the first study of its kind to attempt to define, quantify, and validate the construct of disciplinary literacy.

### Relating findings to existing research

Our results provide an initial step in developing a body of research that defines and validates the multidimensional construct of disciplinary literacy. Our results support and expand upon earlier research in several areas: extending Discourse theory to include disciplinary literacies, developing our understanding of current conceptions of disciplinary literacy, and adding to extant research on literacy in the academic disciplines.

First, our data supports Discourse theory, which views literacy not as tools but as social practices within a community and analyzes how language is used differently in different contexts. For example, our data suggested that ELA teachers were part of an expressive discourse community in which members more frequently practiced the deconstruction of rhetorical and figurative language. If given any particular text, a reader could use the practices of ELA or of history and render different readings. Discourse theory discusses academic language as one language (Snow & Uccelli, 2009), but in fact our study shows that people use language differently across academic contexts. Rather than *academic discourse* in the singular, it may be a better fit to discuss *academic discourses* in the plural. Students need to be made aware of the distinctions of how language is used within each particular academic discipline, which leads to students being able to identify as a member of that community and ultimately to have authority within that discipline.

Second, the data and results expand knowledge about disciplinary literacy. Our findings that literacies are represented in distinct ways within the disciplines align with scholars who argue for disciplinary literacies as an apprenticeship in the discipline (Moje, 2008, 2010; Shanahan & Shanahan, 2008; Shanahan et al., 2011; Zygouris-Coe, 2012) as opposed to a content area literacy approach. The results presented in this research offer support for adolescent literacy instruction occurring through an apprenticeship in the discipline through novice-expert relationships (Ehren et al., 2012). Our results suggest that teachers practice source literacy, analytic literacy, and expressive literacy differently in their academic areas. Given the results of these differentiated practices, teachers can potentially provide opportunities for students to learn the different disciplinary literacy practices. Teachers may have more opportunities to model source literacy in history and social studies than analytic and expressive literacies. In ELA, teachers may be able to provide more focused opportunities to learn expressive literacies. Science and math teachers may provide students more focused opportunities to practice in unique ways with analytic literacy.

Third, our quantitative results support and extend the qualitative findings from previous research on disciplinary literacy. Although Reisman’s (2012) work on history represents a notable exception, available research on this subject has been mostly in the form of qualitative studies (e.g., Park, 2013; Pytash, 2012; Rainey & Moje, 2012; Shanahan & Shanahan, 2008). Shanahan et al. (2011) indicated that there were qualitative differences among literacies in history, chemistry, and mathematics. Our results demonstrated differences among these three areas as well as in the area of ELA and add inferential statistical analysis to demonstrate construct validity to the qualitative research. Rather than each discipline having its own distinct literacy, each discipline operates on a continuum of literacy practices. For example, ELA teachers scored highest for the expressive literacy factor and social studies and history teachers scored highest for source literacy. Our findings also add construct validity to the work of Achugar and Carpenter (2012) and Moje (2007), who claim that the disciplines are not solely about content but also about disciplinary processes, including ways in which knowledge is produced and ways that people read, write, and discuss that knowledge.

## Limitations

Limitations to the present study should be noted. First, although we have convincing support for the current conceptualization of the construct of disciplinary literacy though construct and content validation efforts, we have yet to establish criterion-related validity evidence. This study is the first to our knowledge to quantify disciplinary literacy practices. Prior conceptualizations of disciplinary literacy have found qualitative distinctions by discipline (Shanahan et al., 2011) and increased academic outcomes (Reisman, 2012), yet the collection and validation of such criterion measurements was beyond the scope of the current study and may provide an interesting avenue for future research.

Second, while an extensive literature review process, sampling, and subject matter experts were used to craft the scale items from four targeted disciplines, addressing all disciplines was outside the scope of this study. Our evidence does not indicate whether the current scale would be generalizable to a population of teachers outside the four studied disciplines (e.g., art teachers). Likewise, our participants comprised sixth—twelfth grade teachers because that is when the Common Core State Standards specify literacy standards by discipline. Although disciplinary literacy practices occur at the lower grades, the inclusion of elementary school teachers was beyond the scope of this study.

Third, while we did not use stratified random sampling, we argue that our population is an approximation of a stratified sample. Our sample was approximately half middle school teachers and half high school teachers. In addition, our sample included participants from all four regions used by the US Census (Northeast, South, Midwest, and West; see Fig. 2). We were able to gauge the stratification of our teachers by comparing our sample with national percentages collected by the National Center for Education Information (Feistritzer, 2011). In regard to years of experience, the differences per category ranged from 4 to 10%. For highest degree completed, our percentage included 10% more graduate degrees and 10% less bachelor degrees than the national statistic.

## Implications for policymakers, practitioners, and future research

The results of the study have implications for policymakers, practitioners, and researchers. Our study provides empirical support for policy, practice, and research already underway in addition to providing insight for future applications of disciplinary literacy.

Policymakers would be interested in these results especially in relationship to the Common Core State Standards for literacy in ELA, science and technical subjects, and history and social studies. Results support adoption of literacy standards in ELA, science, and history and social studies and imply that literacy standards in mathematics are warranted. In the future, existing assessments might be improved by making more appropriate and sensitive measures, accounting for the different literacies employed in the disciplines. We do not dismiss the importance of content knowledge, agreeing with Moje (2007), who states, “Reading depends heavily on the content knowledge one brings to a text” (p. 9). Rather, we assert the importance of disciplinary literacy practices and support measurement to improve learning.

For practitioners, the results can help inform the current content area or disciplinary literacy debate (e.g., Collin, 2014; Heller, 2010; Moje, 2010; Monte-Sano et al., 2017), supporting subject area teachers teaching the literacy practices most strongly related to their discipline. The three-factor model of disciplinary literacy provides implications for metacognitive scaffolds as teachers make literacy practices explicit to students. History teachers need more professional development on reading like a historian, since their pedagogical practice may not have kept pace with Wineburg’s (1991a) work on this topic. More professional development is also needed in science and math to give teachers the meta-knowledge about their discipline-specific literacies. Curricula should be structured as discipline-specific literacies in both sixth—twelfth grade education and teacher education as is already occurring in some settings, including the University of Michigan (Shanahan & Shanahan, 2014).

The results of this study lead to additional questions that need to be addressed in future research. Construct validity is an ongoing process (DeCuir-Gunby, 2008). Since we revised the model by eliminating three items, the resulting model needs to be confirmed with a new study. Research is needed with disciplines outside of the four areas addressed in this study. For example, it would be interesting to explore what types of literacies are involved in the disciplines of economics, art, or business. Would these fields draw heavily on the three literacies identified in the current study or would new literacies emerge? Future research could profile readers and writers by discipline from novice to expert. If we do not expect students to become experts in each discipline, what *should* we expect of elementary students, middle grades students, and high school students? We anticipate that the results can be used to generate and test effective tools that classroom teachers use to guide students through the different literacies practiced within the four targeted disciplines of this study.

## Conclusion

Overall, the current research sheds new light on disciplinary literacy and establishes quantitative justification for future work. For those researchers who are invested in establishing extended theories and practices for disciplinary literacy, our research suggests a promising context for fuller discussions as well as a useful base for guiding subsequent studies. Instruction in disciplinary literacies holds promise for improving adolescent literacy and has the potential to engage students in a community of practice that shares a common language and way of thinking.
